# Trends in knee surgery research in the official journal of the Korean Knee Society during the period 1999–2018: a bibliometric review

**DOI:** 10.1186/s43019-020-00046-3

**Published:** 2020-06-08

**Authors:** Seung-Hwan Park, Kwang-Hwan Jung, Sung-Who Chang, Sung-Min Jang, Ki-Bong Park

**Affiliations:** Department of Orthopedic Surgery, Ulsan University Hospital, University of Ulsan College of Medicine, 877 Bangeojinsunhwan-doro, Dong-gu, Ulsan, 44033 South Korea

**Keywords:** Bibliometrics, Korean Knee Society, Research trend, Topic, Surgery

## Abstract

**Background:**

We applied bibliometric tools to original articles published in the official journal of the Korean Knee Society between 1999 and 2018 to identify their characteristics related to knee surgery and to examine the changes in research trends in the last 20 years.

**Methods:**

Over a 20-year period, 579 original articles were published in the journal *Knee Surgery and Related Research* (*KSRR*). We analyzed the title, keywords, and abstract of the article to analyze the research topics and assigned original articles to seven surgical categories as follows: total knee arthroplasty (TKA), unicompartmental knee arthroplasty (UKA), high tibial osteotomy (HTO), arthroscopy, surgery for cruciate ligament, revision surgery, and other surgery. To analyze the trends in research, we divided the study period into two equal parts of 10 years each, examined the percentage of articles in each decade, and analyzed topic trends using the growth rate.

**Results:**

Among the original articles, 86 on the topic of non-surgery were excluded, and 493 original articles related surgical research were included. Articles related to surgery accounted for 85.2% of the total original articles published annually. By period, this was 85.6% in the first half and 84.8% in the late half (*p* = 0.76). A total of 493 original articles related to surgery, with the largest number of TKA-related research at 52.1%. In the study period, the largest increase in the percentage of articles was on the topic of HTO surgery, by 149%. The topics of UKA and revision surgery increased by 95.3% and 33.9%. The topic of TKA increased by 5.9% and the topic of surgery for cruciate ligament decreased by 18.7%. The topic of arthroscopy showed the largest decrease, by 47.6%.

**Conclusions:**

The bibliometric findings of this study suggest that the majority of surgery-related original articles published in *KSRR* during the last 20 years involved research about TKA surgery, and the greatest relative increase over the study period involved research about HTO surgery. The authors expect that the analysis of characteristics and research trends of original articles published in *KSRR* will provide useful information about *KSRR* for future researchers.

## Introduction

Bibliometrics is the branch of library science that uses mathematical and statistical techniques to analyze books, articles, and other documents [1]. A bibliometric study evaluates the performance of each article and provides a comprehensive evaluation of research trends [2, 3]. Therefore, readers can understand the trends in research in a specific field through bibliometric analyses.

Various bibliometric analyses have been performed on articles published in the orthopedics field [4–7]. In addition, there were analyses of articles published in general orthopedic journals [8] or articles focused on the field of specific knee surgeries [9, 10]. However, to date, no bibliometric analysis analyzed the topic of articles published in a specific single journal focusing on knee-related surgery.

*Knee Surgery and Related Research* (*KSRR*) is the official journal of the Korean Knee Society (KKS) and made its debut in September 2011, replacing *The Journal of Korean Knee Society* published since 1989 by the KKS. This journal covers all fields of clinical knee surgery and basic research related to knee surgery.

In the more than 30 years since 1989, no studies have centered on a bibliometric analysis of the characteristics of articles published in *KSRR*. Here, we attempt to build upon existing bibliometric research for this journal. Our focus, however, is on the title and keywords of each article, rather than the authors, the affiliation, or the citation index.

When performing a bibliographic analysis of articles, the contents analyzed are bibliometric parameters used commonly and the topic of each article, identified using the titles and keywords of articles. The article’s title presents what was studied, how this was done, and what are the major results. Therefore, a concise title is informative, attractively conveys the main topic, and highlights the importance of the study [11]. In addition, keywords indicate the author’s opinion of the three to six most important words in their articles. Therefore, the title and keywords are an important clue to grasp the topic of the article [12].

Analysis of research topics using titles and keywords helps readers to know not only current research trends in a specific field, but also past research trends [13]. Bibliographic analysis of the topic of the articles enables one to know the most frequent research topics in a specific journal, and the changes in frequency of a specific topic as times change.

The purpose of this study was to evaluate original articles published in *KSRR* during the last 20 years and identify the characteristics of articles related to knee surgery and the changes in research trends in the field of knee surgery.

## Materials and methods

### Data collection

A search of the literature was conducted using an online database to identify all literature published in *KSRR* from 1 January 1999 to 31 December 2018. Two authors classified all literature by the type of publication and reviewed the literature to clarify the topic of articles, screening by title, keywords, and abstract of the article. If the clarification of the topic was ambiguous in the analysis process, the authors additionally analyzed the full text. We included all original articles in the analysis and excluded other types of publications, such as review articles, case reports, editorials, technical notes, and letters to the editor.

### Categorization

We assigned original articles to seven surgical categories as follows: total knee arthroplasty (TKA), unicompartmental arthroplasty (UKA), high tibial osteotomy (HTO), arthroscopic surgery, surgery for cruciate ligament, revision surgery, and other surgery. ‘Arthroscopic surgery’ included surgeries using arthroscopy—meniscectomy, meniscal repair, cyst decompression, and pull-out suture for avulsion fracture. ‘Surgery for cruciate ligament’ included arthroscopic reconstruction or repair for cruciate ligaments. In addition, we calculated the proportion of original articles for each surgery.

### Analysis strategy for research trends

To analyze the trends in research, we divided the 20-year study period into two equal parts of 10 years each: 1999–2008 (first half) and 2009–2018 (late half). Since the total number of original articles published each year is different, the percentage of articles for each category was calculated. We calculated the percentage of each category in each half by dividing the number of articles about each category by the number of articles about all categories in each half. In addition, we evaluated the order of each category in each half and the growth rate of each category in the last half was calculated. We calculated the growth rate by dividing the difference in the percentage of each category in the two periods by the percentage in the first half.

### Similarity analysis between the research and surgery utilization trend in Korea

To determine the similarity between the research trend and the surgical trend, we evaluated TKA, UKA, HTO, arthroscopic surgery, surgery for cruciate ligament, and revision surgery utilization in Korea between 2009 and 2018 using the Health Insurance Review and Assessment Service (HIRA) of Korea database. All patients with the procedural codes for primary TKA (N2072, N2077), primary UKA (N2712, N2717), tibial and/or fibular osteotomy with internal fixation (N0304, N0307), arthroscopic surgery (N0821, N0826, N0822, N0827, N0823, N0828, N0824, N0829), surgery for cruciate ligament (N0880, N0881, N0890), and revision TKA (N3712, N2717, N3722, N 3727) were identified.

### Statistical analysis

We performed the paired *t* test for comparison between the values of the two periods, first half versus late half, and used the IBM SPSS Statistics program (version 20.0; IBM Corp., Armonk, NY, USA) for all analyses. The threshold for statistical significance was *p* < 0.05.

## Results

During the 20-year period between January 1999 and December 2018, there were a total of 579 original articles published in *KSRR*. Of these, we excluded 86 publications that were articles on the topic of non-surgery. Our final dataset comprised 493 original articles.

### Number of original articles

**Fig. 1:**
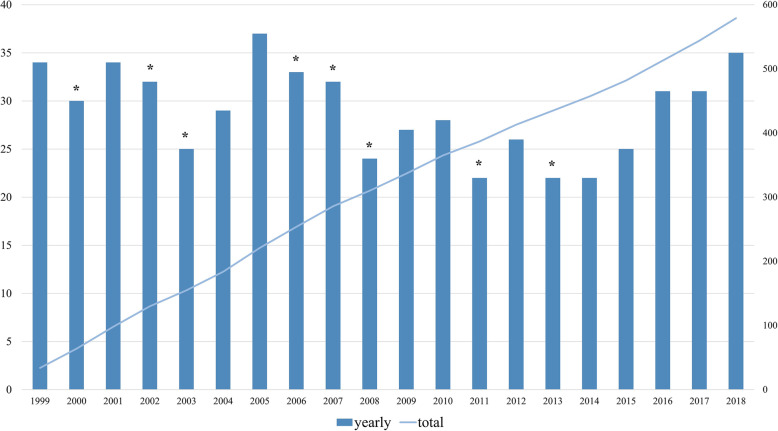
Number of original articles published in *Knee Surgery and Related Research* by year. *Year in which the number of published original articles in the year has decreased from the previous year. Straight line represents all original articles published during the study period

Figure 1 shows the cumulative and yearly distribution of original articles published by *KSRR* in the last 20 years. The average number of published articles per year was 29. In the first half, 31 articles were published annually on average, but in the late half, on average, 26.9 articles were published annually, which was not increased (*p* = 0.05). Analyzing with the average year by year, 8 years had negative increment values in the number of total articles and surgery-related articles since 1999.

### Percentage of articles related to surgery

**Fig. 2:**
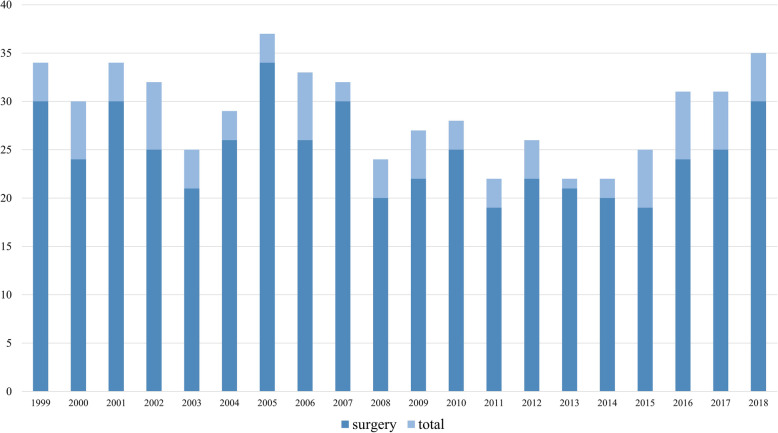
Number of original articles related to surgery over the study period by year

**Fig. 3:**
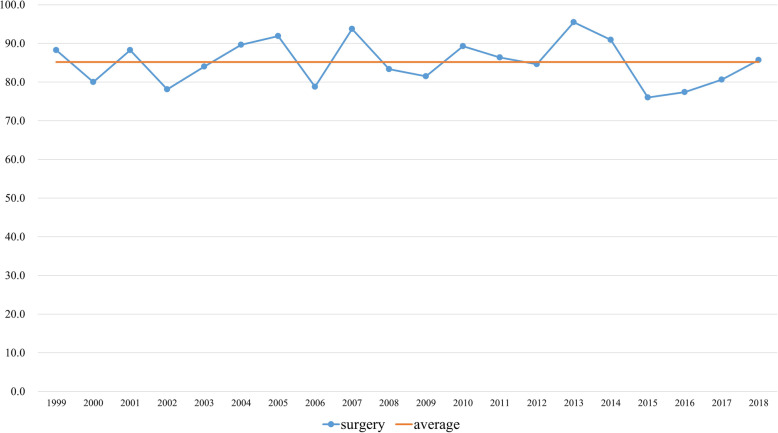
Average proportion of original articles related to surgery over the study period by year

The number of articles related to surgery increased by an average of 25 articles each year (Fig. 2). Articles related to surgery accounted for 85.2% of the total original articles published annually (Fig. 3). By period, the proportion of articles related to surgery was 85.6% in the first half and 84.8% in the late half (*p* = 0.76).

### Percentage of categories by surgery

**Fig. 4:**
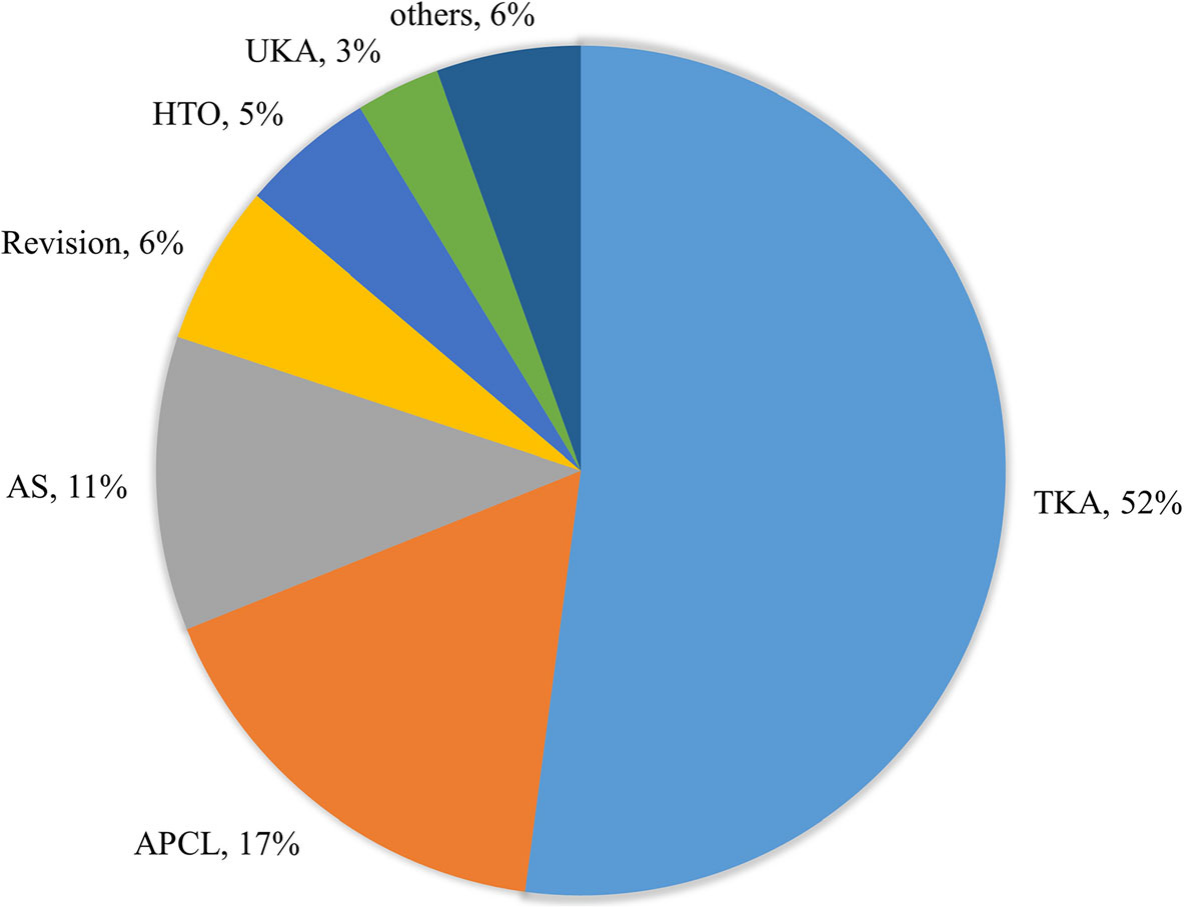
Pie chart demonstrating the percentage of categories by surgery. *APCL* anterior or posterior cruciate ligament, *AS* arthroscopy, *HTO* high tibial osteotomy, *TKA* total knee arthroplasty, *UKA* unicompartmental knee arthroplasty

Figure 4 shows the percentage of categories by surgery. Articles in the TKA category were the highest (52.1%, *n* = 257), followed by surgery for cruciate ligament (16.8%, *n* = 83), arthroscopy (11.2%, *n* = 55), revision surgery (6.1%, *n* = 30), HTO (5.1%, *n* = 25), UKA (3.2%, *n* = 16), and other surgery (5.5%, *n* = 27). About four-fifths of articles covered the categories of TKA, surgery for cruciate ligament, and arthroscopy (80.1%).

### Trends of categories by surgery in each period

In the first half, the most common category by surgery was TKA research (50.8%), followed by surgery for cruciate ligament (18.4%), arthroscopy (14.3%), revision surgery (5.3%), HTO (3%) and UKA (2.3%) (Table 1). However, in the late half, the percentage of HTO research increased, exceeding the percentage of revision research (7%), and became the same as the percentage of arthroscopy research (7.5%).

**Table 1:** Growth rate of articles related to each surgery published in the official journal of Korean Knee Society for the last 20 years

Category	First half (1999–2008), *n* (%)	Late half (2009–2018), *n* (%)	Growth rate (%)
HTO	8 (3)	17 (7.5)	+ 149
UKA	6 (2.3)	10 (4.4)	+ 95.3
Revision surgery	14 (5.3)	16 (7)	+ 33.9
TKA	135 (50.8)	122 (53.7)	+ 5.9
APCL	49 (18.4)	34 (15)	– 18.7
AS	38 (14.3)	17 (7.5)	– 47.6
Other surgery	16 (6)	11 (4.8)	– 19.4

*APCL* anterior or posterior cruciate ligament, *AS* arthroscopy, *HTO* high tibial osteotomy, *TKA* total knee arthroplasty, *UKA* unicompartmental knee arthroplasty

### Topic trends using the growth rate

**Fig. 5:**
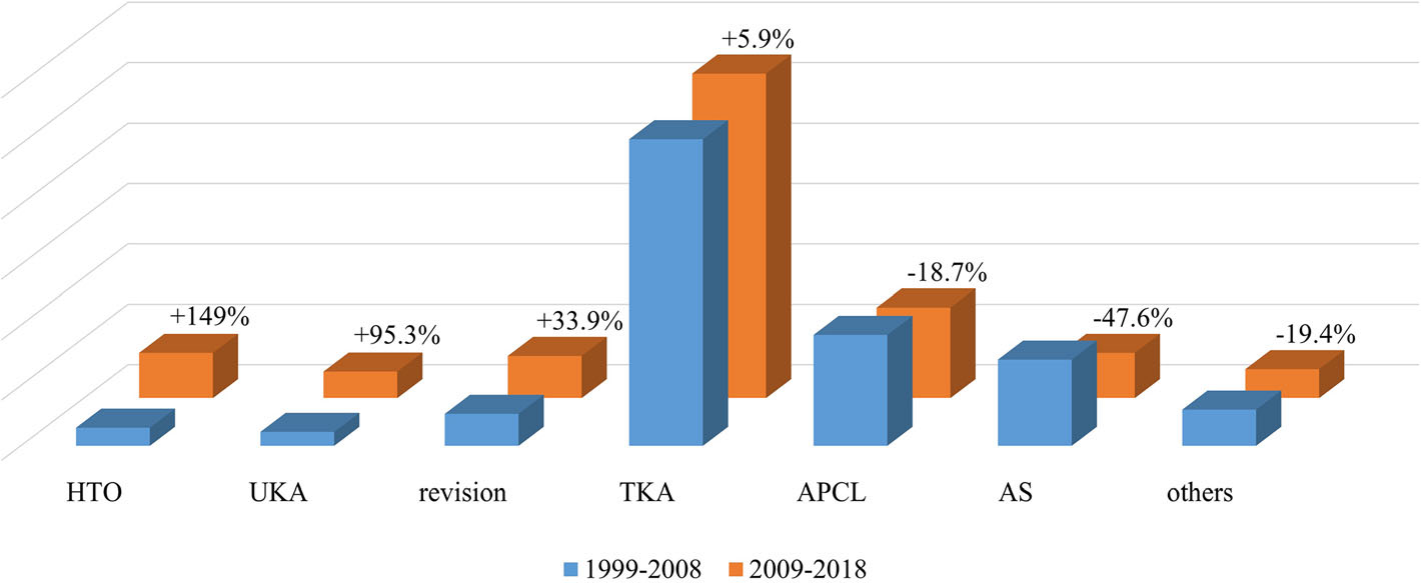
Proportion of original articles by category for the first half and the late half. Values (%) indicate the growth rate of each category. *APCL* anterior or posterior cruciate ligament, *AS* arthroscopy, *HTO* high tibial osteotomy, *TKA* total knee arthroplasty, *UKA* unicompartmental knee arthroplasty

Figure 5 shows the proportion of original articles by category for the first half and the late half. HTO research increased the most during the study period, with a growth rate of 149%. Then, the amount of research for UKA, revision surgery, and TKA increased by 95.3%, 33.9%, and 5.9% respectively. In addition, the topic of surgery for cruciate ligament decreased by 18.7%. Finally, arthroscopy research decreased the most during the study period, by 47.6%. Figure 5 shows the increasing and decreasing trends for each category by surgery during the study period.

### Actual number of surgeries performed in Korea and the growth rate

Table 2 presents the number of surgeries by year obtained from the HIRA and the growth rate for the late half (2014–2018) compared to the first half (2009–2013). According to the category of surgery performed in Korea from 2009 to 2018, arthroscopy was the highest with 863,799 cases, followed by TKA (548,003 cases), surgery for cruciate ligament (147,199 cases), and HTO (93,947 cases). The growth rate by surgery category was the highest in HTO (102.6%), followed by UKA (57.9%), TKA (33.3%), revision surgery (18.7%), surgery for cruciate ligament (4.6%), and arthroscopy (2.4%).

**Table 2:** Actual number and growth rate of surgery performed in Korea between 2009 and 2018

Category	Total number	First half (2009–2013) (*n*)	Late half (2014–2018) (*n*)	Growth rate (%)
HTO	93,947	31,047	62,900	102.6
UKA	31,902	12,372	19,530	57.9
TKA	548,003	234,891	313,112	33.3
Revision surgery	24,895	11,383	13,512	18.7
APCL	147,199	71,952	75,247	4.6
AS	863,799	426,850	436,949	2.4

*APCL* anterior or posterior cruciate ligament, *AS* arthroscopy, *HTO* high tibial osteotomy, *TKA* total knee arthroplasty, *UKA* unicompartmental knee arthroplasty

## Discussion

This study conducted a bibliometric analysis of knee surgery-related original articles published in *KSRR* over the last 20 years and analyzed the proportion of articles related to surgery and the trends in topics of research.

The average number of articles published in the last half did not increase compared to the first half (*p* = 0.05). However, since *KSRR* published a new type of publication—a review article—since 2009, it is necessary to understand whether the composition of the publication in the journal has changed in order to interpret the results of this study.

As the name suggests, *KSRR* published articles about ‘research related to knee surgery’, and the results of this study showed that the original articles relating to surgery accounted for about 80.5% of the total original articles. In addition, since there was no difference of proportion year by year and in each decade, we may presume that the future trend will be similar.

The *KSRR* analysis of this study shows that TKA, surgery for cruciate ligament, and arthroscopy accounted for 80% of all topics of articles published in the last 20 years. Similarly, in the HIRA database analysis, we found that these same surgical categories accounted for 91.2% of all surgeries performed in the last 10 years.

In the analysis using the HIRA database, the category of surgery with the highest increase in Korea from 2009 to 2018 was HTO (102.6%). Although the study period was different, it is interesting that the increase in HTO-related research was the largest in the last 20 years, like the increase of actual surgeries was the largest in HTO in the last 10 years.

Over the last decade, the primary and revision TKA rates increased by 33.3% and 18.7%, respectively. However, we reported that revision-related research increased by 33.9%, while TKA-related research increased by 5.9%. This means that publication of research about revision surgery has markedly increased compared to the topic of TKA research.

Compared to the first half, the proportion of research on surgery for cruciate ligament and arthroscopy declined in the late half, but both topics still accounted for 22.5% of surgery-related research in the late half. Similarly, in the analysis using the actual number of surgeries performed, surgery for cruciate ligament and arthroscopy showed a relatively low growth rate of about 2.7% in the late 5 years compared to the first 5 years. However, surgery for cruciate ligament and arthroscopy accounted for about 55.6% of all surgeries performed in the late 5 years, indicating that the two surgeries still accounted for a significant proportion in the field of knee surgery.

This study shows that the proportion of the research for arthroscopy or surgery for cruciate ligament was relatively reduced in the late half compared to the first half. The Korean Orthopaedic Association, to which the KKS belongs, has a subspecialty society that deals only with the arthroscopic field. In addition, since the arthroscopic society has an official journal, we cannot exclude the possibility of submission to that journal. In addition, the possibility that arthroscopy or surgery for cruciate ligament research can be published in academic journals of other societies should be fully considered. Therefore, we should not interpret that the absolute number of research related to arthroscopy or surgery for cruciate ligament has decreased. However, since the possibility of submitting research on other surgical-related topics to other journals is the same, it is possible to interpret meaningfully the trends in the proportion of topics in a single journal.

The readers must view the results of this study in the light of the following limitations. Firstly, this study is an analysis of research trends. Therefore, the results of this study do not reflect the actual amount of surgery performed over 20 years. Secondly, the results of this study cannot reflect global research trends because we analyzed only articles published in a single journal. Thirdly, only one author screened all articles by title, keywords, and abstract during the exclusion and categorization stages, which increases the risk of bias, although we attempted to mitigate this with clear predefined exclusion and category criteria. Fourthly, the arthroscopy-related articles include a variety of disease groups and various surgical methods, which limits the inferiority of analysis as an analysis target compared to other single surgical method-related articles. Finally, we may overlook finer details in research trends on each topic related to knee surgery from 1999 to 2018 because of performing only an analysis based on the decades.

## Conclusion

The majority of surgery-related original articles published in *KSRR* during the last 20 years involved research about TKA surgery, and the greatest relative increase over the study period involved research about HTO surgery. The authors expect that analysis of characteristics and research trends of original articles published in *KSRR* will provide useful information about *KSRR* for future researchers.

## Data Availability

Not applicable.
